# Clinicopathological characteristics and risk factors in elderly patients with biopsy-proven IgA nephropathy

**DOI:** 10.1080/0886022X.2022.2087527

**Published:** 2022-06-29

**Authors:** Jiaxing Tan, Xinyao Luo, Jiaqing Yang, Nuozhou Liu, Zheng Jiang, Yi Tang, Wei Qin

**Affiliations:** a Division of Nephrology, Department of Medicine, West China Hospital, Sichuan University, Chengdu, Sichuan, China; b West China School of Medicine, Sichuan University, Chengdu, Sichuan, China

**Keywords:** Immunoglobulin A nephropathy, observational study, advanced age, immunosuppressive therapy, renin-angiotensin system

## Abstract

**Background:**

Immunoglobulin A nephropathy (IgAN) has been well studied among young people, but few data on clinicopathological characteristics, treatment response and outcomes for elderly IgAN patients are available.

**Methods:**

A cohort study of elderly IgAN patients was performed. The combined endpoints of renal outcome were a 50% decline in eGFR compared with the time of renal biopsy, end-stage kidney disease and/or death. Risk factors associated with poor renal outcomes were then determined. The benefits of immunosuppressant therapies were also evaluated by Kaplan-Meier survival curve analysis.

**Results:**

This study ultimately included 126 elderly patients with IgAN. Comparison between the endpoint and non-endpoint groups indicated that patients with poor outcomes had more severe clinical features, such as worse kidney function, severe hematuria and lower albumin levels. Cox regression analysis indicated that age (HR 1.15, 95% CI 1.02–1.29, *p* = 0.021), male gender (HR 9.71, 95% CI 1.00–97.56, *p* = 0.050), and urine red blood cells (HR 1.003, 95% CI 1.000–1.006, *p* = 0.029) were independent risk factors for poor renal outcome in elderly IgAN patients. To explore possible reasons accounting for the predictive value of age and sex, patients were divided into two groups based on these two variables. Patients in the geriatric group had lower serum albumin, estimated glomerular filtration rate, hemoglobin and aspartate aminotransferase levels than those in the quinquagenarian group. Male patients tended to have higher hemoglobin, higher alanine aminotransferase, and lower triglycerides and cholesterol levels than female patients. To investigate different treatment responses, patients were classified into two groups depending on treatment strategies (renin-angiotensin system inhibitors and immunosuppressive therapy), and the survival analysis indicated no significant difference in kidney outcome between the two groups (*p* > 0.05). This result still holds after adjusting for age, sex, eGFR, hematuria, and proteinuria.

**Conclusion:**

Advanced age, male, and hematuria might be independently associated with poor kidney outcomes in elderly patients with IgAN. Immunosuppressive therapy might confer no overall benefit to older IgAN patients.

## Introduction

Immunoglobulin A nephropathy (IgAN) is the most common form of primary glomerulonephritis worldwide [1,2]. It has been reported that approximately 20–40% of IgAN patients have developed end-stage kidney disease (ESKD) within 20 years of disease onset [3]. While the disease may affect people at any age, it occurs more frequently in patients aged 20–50 years old and is less common in the elderly [2,4]. Therefore, many studies only investigated young IgAN patients, and few data on clinical features, treatment and outcomes were found among its counterpart.

Identifying the risk factors for the disease has an important guiding role in treatment planning. To date, there are some well-recognized risk factors associated with the progression of poor renal outcomes in patients with IgAN, including proteinuria of more than 1 g/24h, sustained hypertension and kidney insufficiency at onset [5–7]. However, the impact of hematuria, aging and sex on the progression of IgAN remains controversial [8–11]. Meanwhile, most of these relevant studies were specifically designed for the evaluation of younger IgAN patients under the age of 50 years old, but no consensus was reached in older patients [9]. Considering the physiological age-related decline in kidney function and other chronic diseases in elderly individuals, it is possible that IgAN patients diagnosed at an advanced age may have other predictive factors than their younger counterparts.

Guideline by the Kidney Disease: Improving Global Outcomes (KDIGO) recommended angiotensin-converting enzyme inhibitor (ACEI)/angiotensin receptor blocker (ARB) as the first-line therapy in IgAN patients, but the effects of immunosuppression and corticosteroids are still in dispute [12]. Much attention has been focused on whether additional immunosuppressive therapy confers more advantages than supportive approaches alone, and indeed, several clinical trials have demonstrated the protective effect of corticosteroids, especially for patients with proteinuria of more than 1 g/24h [13–16]. Nevertheless, consistency has not been reached. As highlighted by a recent STOP-IgAN clinical trial, the addition of steroids and immunosuppressants did not confer better renal outcomes, and adverse effects caused by generalized immunosuppression were obviously observed in the TESTING trials [15,17]. At the same time, there are limited published studies specifically designed to examine the impact of immunosuppressants on elderly patients, so further evaluation of therapeutic strategies for that age group is needed to address the evidence gap.

Therefore, we conducted a prospective cohort analysis of elderly IgAN patients (age ≥50 years) to fully elucidate the details of clinicopathological characteristics, identify the risk factors for IgAN progression to ESKD and compare the therapeutic effects of different immunosuppressive therapies in the elderly population.

## Methods

### Subjects and follow-up

Patients diagnosed with IgAN between May 2009 and September 2019 at West China Hospital of Sichuan University were prospectively recruited for our study. The diagnosis was confirmed by renal biopsies [18]. The inclusion criteria applied were as follows: (1) patients confirmed to have IgAN by renal biopsy; (2) patients who were followed up for at least 12 months or who reached a predefined endpoint within 12 months; and (3) patients ≥ 50 years old. The exclusion criteria were as follows: (1) patients with systemic diseases, such as diabetes mellitus, systemic lupus erythematosus (SLE), Henoch-Schonlein purpura (HSP), liver cirrhosis or disorder of liver function, and malignancy; (2) patients without complete clinical data. Written informed consent for participation was obtained from all patients or their legal guardians before enrollment. This study complies with the Helsinki Declaration and was approved by the Ethics Committee of West China Hospital of Sichuan University (approval number 2019-33).

Most patients were scheduled for regular follow-up reexaminations in the out-patient department of West China Hospital of Sichuan University for at least 12 months or shorter if they reached the predefined study endpoints. Some patients who did not have regular visits to our medical center were followed up by phone calls.

### Data collection

Clinical and laboratory data including age, sex, systolic blood pressure (SBP), diastolic blood pressure (DBP), serum creatinine (Scr), serum albumin, 24-h proteinuria (UPRO), hematuria level (URBC), hemoglobin, alanine aminotransferase (ALT), aspartate aminotransferase (AST), estimated glomerular filtration rate (eGFR), triglycerides, and cholesterol, were collected at the time of renal biopsy. The eGFR was calculated according to the CKD-EPI (CKD Epidemiology Collaboration) formula [19]. Hypertension was defined as blood pressure >140/90 mmHg [20]. Anemia was defined as hemoglobin <120 g/L in men and <110 g/L in women. Hematuria was defined as urine RBC >5/HPF using the standard manual examination of urine sediment.

Renal biopsies were evaluated by at least two experienced pathologists and two nephrologists who were blinded to the patients’ clinical condition. When discrepancies or doubts in diagnosis existed, these were submitted to a higher-level pathologist for evaluation. All researchers were blinded to the pathological evaluation. The pathological lesions were graded according to the updated Oxford classification [21].

### Outcomes

The combined endpoints of kidney outcome were a 50% decline in eGFR compared with the time of renal biopsy, ESKD and/or death. ESKD was defined as eGFR < 15 mL/min/1.73m^2^, treatment by dialysis, or requiring kidney replacement treatments.

### Groups and treatments

First, patients were classified into two groups based on whether they had reached the kidney endpoints to investigate the potential predictive factors, which were further confirmed by Cox analysis and subgroup analysis. Patients were subsequently divided into several subgroups based on the presence of hematuria, level of eGFR (eGFR > 60 mL/min/1.73m^2^ vs. eGFR≤ 60 mL/min/1.73m^2^) and proteinuria (proteinuria < 1 g/d vs. proteinuria ≥ 1 g/d). In addition to clinical factors, age (50–59 years old vs. ≥60 years old) and sex (male vs. female) were also considered to investigate the effect of demographic data on kidney prognosis.

Therapeutic regimens were determined by both patients and attending doctors according to clinical and pathological characteristics. Researchers were not involved in the formulation of treatment plans. Patients were classified into two groups: renin-angiotensin system inhibitors (RASi) and immunosuppressive therapy (IST), which was composed of a prednisone (Pred) group and an immunosuppressant (ISA) group. The RASi group was given a full dose of angiotensin-converting enzyme inhibitors (ACEIs) or angiotensin receptor blockers (ARBs). The Pred group received optimal supportive care combined with corticosteroids (0.5–1 mg/kg daily and tapered down within 6–8 months). Patients in the ISA groups received supportive care plus corticosteroids (dosage as mentioned above) and immunosuppressants (mycophenolate mofetil, cyclophosphamide, or azathioprine). Patients in all groups received standard medical care. Since this was an observational study, the baseline characteristics of the patients were not well homogenous. Therefore, we adjusted for age, sex, eGFR, hematuria, and proteinuria for subgroup analysis to minimize the impact of different baseline characteristics.

### Statistical analyses

Categorical variables are presented as frequencies (percentages) and were analyzed using chi-squared tests or Fisher's exact test. Continuous variables were expressed as the mean ± standard deviation (SD) or median (interquartile range) and were compared by t test, one-way ANOVA or Kruskal-Wallis Test. The cumulative probability of progressing into kidney outcomes was estimated by Kaplan-Meier survival analysis, and survival curves were compared using the log-rank test. Univariate and multivariate Cox proportional hazard models were used to assess the effect of demographic and clinicopathological features on kidney endpoints. Two-tailed *p* < 0.05 was considered statistically significant. All statistical analyses were performed using IBM SPSS statistics 26.0 software.

## Results

### Baseline characteristics

**Figure 1.**
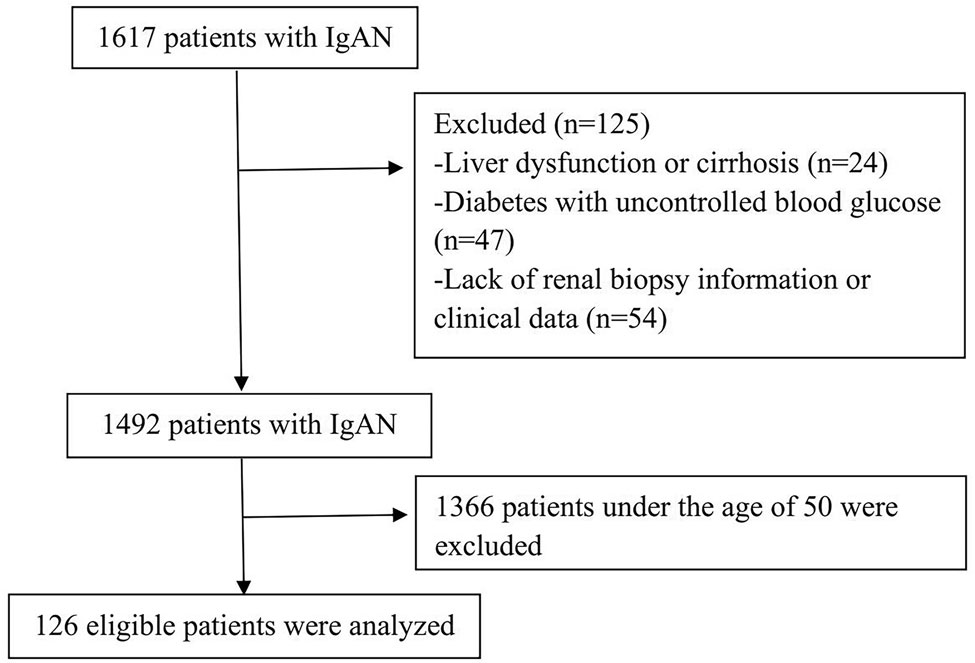
Study flow chart. The patients were recruited into the study according to the flow chart shown.

The study flow chart is presented in Figure 1. A total of 1617 patients diagnosed with IgAN in our medical center between May 2009 and September 2019 were primarily included. Among these patients, 125 patients were excluded according to the following exclusion criteria: under the age of 50 (*n* = 1366), liver dysfunction or cirrhosis (*n* = 24), diabetes with uncontrolled blood glucose (*n* = 47), and lack of renal biopsy information or clinical data (*n* = 54). The study finally included 126 eligible patients. The mean age of this cohort was 57.12 ± 6.24 years, with a predominantly female gender(61%). Patients were followed up for 54.4 ± 23.9 months on average. Of the patients, 35 patients (27.8%) had a history of hypertension and anemia was observed in 15 patients (11.9%).

Patients were divided into two groups according to whether subjects progressed to the endpoint of kidney outcome: the endpoint group (*n* = 12) and the non-endpoint group (*n* = 114). The demographic and clinicopathological data of each group are shown in Table 1. The age at diagnosis was significantly higher in patients who progressed to the endpoint than in those who did not (62.83 ± 7.92 vs. 56.52 ± 5.76, *p* = 0.001). Moreover, patients over the age of 60 and male patients seemed to be more likely to progress to ESKD. Comparison between the two groups also indicated that patients with poor outcomes had more severe clinical features, such as higher levels of U-RBC (17 [5–77] vs. 86 [59–323], *p* = 0.006) and SCr (171.20 ± 71.09 vs. 106.36 ± 57.08, *p* < 0.001), and lower albumin (32.18 ± 6.91 vs. 37.31 ± 6.45, *p* = 0.010) and eGFR (44.37 ± 24.98 vs. 68.89 ± 25.68, *p* = 0.002) levels. However, there were no intergroup differences among some common clinical indexes, such as rates of hypertension (25.0% vs. 28.1%, *p* = 1.000) and anemia (8.3% vs. 12.3%, *p* = 1.000), and levels of proteinuria (2.81 ± 1.59 vs. 2.69 ± 2.83, *p* = 0.887). Regarding renal pathologic lesions, patients in the endpoint group presented with a significantly higher frequency of segmental glomerulosclerosis (8.3%/91.7% vs. 46.5%/53.5%, *p* = 0.013) and tubular atrophy/interstitial fibrosis (50.0%/50.0%/0% vs. 86.8%/12.3%/0.9%, *p* = 0.005) than those in the non-endpoint group, while there was no marked difference in the presence of mesangial proliferation (0%/100% vs. 91.2%/8.8%, *p* = 0.068), endocapillary proliferation (8.3%/91.7% vs. 46.5%/53.5%, *p* = 0.596), or crescents (75.0%/16.7%/8.3% vs. 77.2%/20.2%/2.6%, *p* = 0.412).

**Table 1.** Clinicopathological manifestations and laboratory data of the older IgAN patients at baseline.

Variables	All (*n* = 126)	Control (*n* = 114)	Case (*n* = 12)	*p* values
Age at diagnosis (years)	57.12 ± 6.24	56.52 ± 5.76	62.83 ± 7.92	0.001***
Age ≥ 60 (%)	39 (31.0)	32 (28.1)	7(58.3)	0.047*
Female (%)	61 (48.4)	60 (52.6)	1(8.3)	0.004**
SBP (mmHg)	133.74 ± 17.55	133.03 ± 16.75	140.50 ± 23.71	0.161
DBP (mmHg)	84.41 ± 12.42	84.45 ± 12.37	84.08 ± 13.44	0.924
Hypertension (%)	35 (27.8)	32 (28.1)	3 (25.0)	1.000
Proteinuria (g/24h)	2.70 ± 2.73	2.69 ± 2.83	2.81 ± 1.59	0.887
U-RBC (/HP)	21 (6–112)	17 (5–77)	86(59–323)	0.006**
Albumin(g/L)	36.82 ± 6.64	37.31 ± 6.45	32.18 ± 6.91	0.010**
SCr (umol/L)	112.54 ± 61.28	106.36 ± 57.08	171.20 ± 71.09	<0.001***
e-GFR (ml/min/1.73m^2^)	66.55 ± 26.52	68.89 ± 25.68	44.37 ± 24.98	0.002**
UA (umol/L)	393.69 ± 98.29	387.29 ± 95.89	454.50 ± 104.22	0.024*
Hemoglobin (g/L)	129.30 ± 20.37	129.92 ± 19.67	123.42 ± 26.44	0.295
Anemia (%)	15 (11.9)	14 (12.3)	1 (8.3)	1.000
Triglyceride (mmol/L)	2.12 ± 1.36	2.19 ± 1.40	1.45 ± 0.50	0.071
Cholesterol (mmol/L)	5.36 ± 1.30	5.38 ± 1.31	5.14 ± 1.20	0.535
ALT (IU/L)	23.52 ± 13.22	23.14 ± 13.11	27.17 ± 14.28	0.318
AST (IU/L)	24.70 ± 9.13	24.26 ± 9.08	28.83 ± 9.07	0.099
M0/M1 (%)	27/99 (21.4/78.6)	27/87 (23.7/76.3)	0/12 (0/100)	0.068
E0/E1 (%)	116/10 (92.1/7.9)	104/10 (91.2/8.8)	12/0 (100/0)	0.596
S0/S1 (%)	54/72 (42.9/57.1)	53/61 (46.5/53.5)	1/11 (8.3/91.7)	0.013**
T0/1/2 (%)	105/20/1 (83.3/15.9/0.8)	99/14/1 (86.8/12.3/0.9)	6/6/0 (50.0/50.0/0)	0.005**
C0/1/2 (%)	97/25/4 (77.0/19.8/3/2)	88/23/3 (77.2/20.2/2.6)	9/2/1 (75.0/16.7/8.3)	0.412

*SBP:* systolic blood pressure; *DBP:* diastolic blood pressure; *U-RBC:* the count of uric red blood cell; *SCr:* serum creatinine; *e-GFR:* estimated glomerular filtration rate; *UA:* uric acid; *ALT:* alanine aminotransferase*; AST:* aspartate aminotransferase*;* *M:* mesangial proliferation; *E:* endocapillary proliferation; *S:* segmental glomerulosclerosis; *C:* crescents; *T:* tubular atrophy/interstitial fibrosis.

*Stands for *p* < 0.05; **stands for *p* ≤ 0.01; ***stands for *p* ≤ 0.001.

### Risk factors for kidney outcomes

Univariate and multivariate Cox proportional hazard regression models were performed to analyze the relationship between clinicopathological parameters and kidney outcomes (Table 2). A multivariate Cox regression model indicated that age (HR 1.15, 95% CI 1.02–1.29, *p* = 0.021), male (HR 9.71, 95% CI 1.00–97.56, *p* = 0.050), segmental glomerulosclerosis (HR 42.45, 95% CI 1.36–1325.33.12, *p* = 0.033), tubular atrophy or interstitial fibrosis (HR 4.35, 95% CI 1.12–16.89, *p* = 0.034) and U-RBC (HR 1.003, 95% CI 1.000–1.006, *p* = 0.029) were independent risk factors for kidney survival.

**Table 2.** Prediction of renal outcomes in the older IgAN was carried out by Cox-regression analysis.

	Univariate analysis		Multivariate analysis	
	HR (95%CI)	*p*	HR (95%CI)	*p*
Age (years)	1.13 (1.04–1.23)	**0.003**	1.15 (1.02–1.29)	**0.021**
Male (vs. Female)	10.54 (1.34–81.80)	**0.024**	9.71 (1.00–97.56)	**0.050**
S1 (vs. S0)	9.49 (1.23–73.60)	**0.031**	42.45 (1.36–1325.33)	**0.033**
T1/T2 (vs. S0)	6.55 (2.11–20.37)	**0.001**	4.35 (1.12–16.89)	**0.034**
Proteinuria (g/24h)	1.00 (0.83–1.21)	0.998	0.79 (0.56–1.12)	0.189
U-RBC (/HP)	1.002 (1.000–1.003)	**0.017**	1.003 (1.000–1.006)	**0.029**
eGFR (ml/min/1.73m^2^)	0.96 (0.93–0.99)	**0.003**	0.98 (0.94–1.01)	0.205

Bold values are statistically significant difference.

*M:* mesangial proliferation; *E:* endocapillary proliferation; *S:* segmental glomerulosclerosis; *T:* tubular atrophy or interstitial fibrosis; *C:* crescents; *U-RBC:* the count of uric red blood cell; *ALB:* albumin; *e-GFR:* estimated glomerular filtration rate.

**Figure 2.**
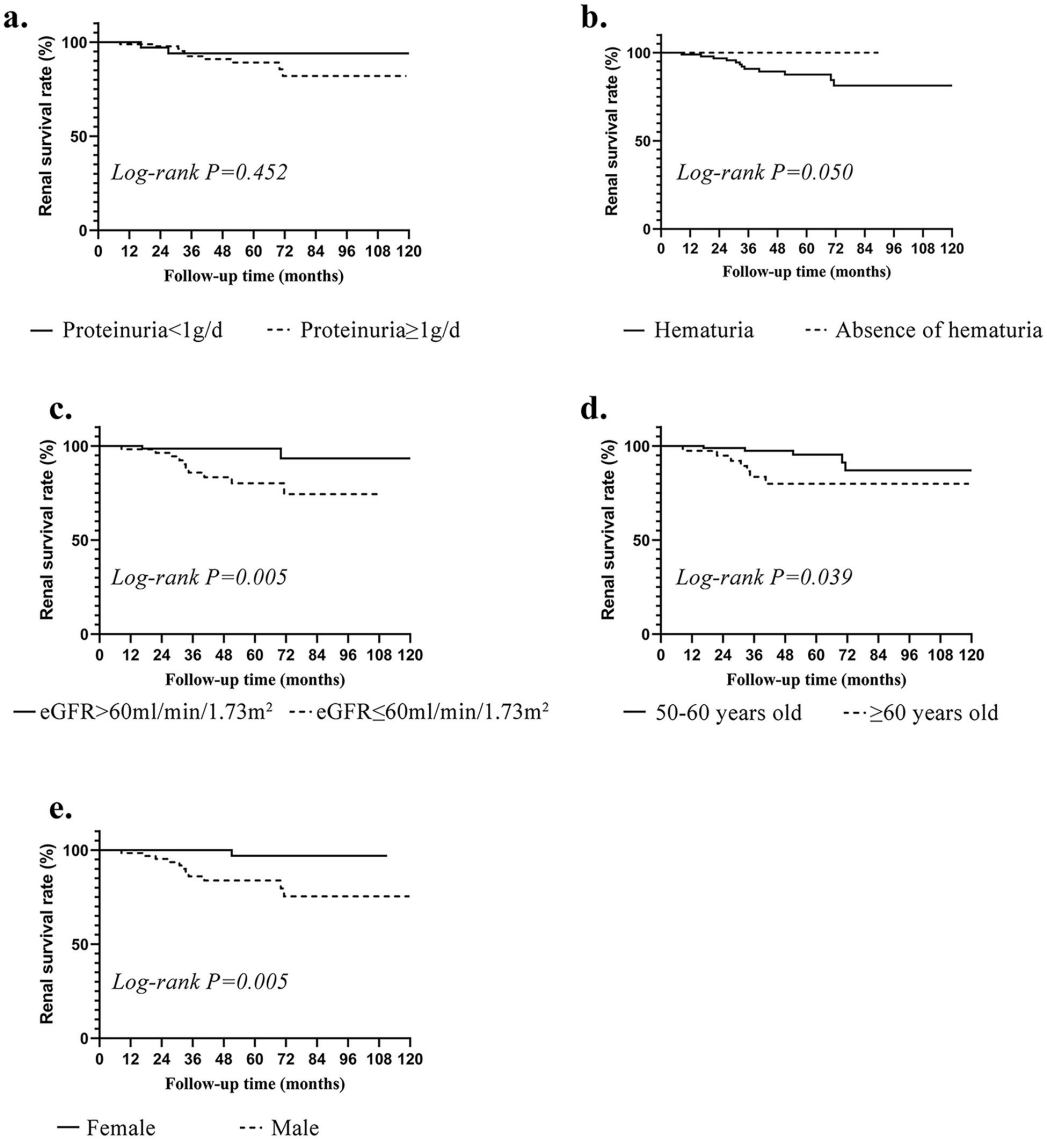
Kaplan-Meier curves of kidney survival according to demographic and clinical features. (a) Stratified by proteinuria (proteinuria < 1g/d vs. proteinuria > 1g/d); (b) Stratified by hematuria (hematuria vs. absence of hematuria); (c) Stratified by level of eGFR (eGFR > 60ml/min/1.73m^2^ vs. eGFR < 60ml/min/1.73m^2^); (d) Stratified by age (50–60 years old vs. ≥ 60 years old); (e) Stratified by gender (female vs. male). eGFR, estimated glomerular filtration rate.

The Kaplan-Meier survival analysis was carried out according to the demographic and clinical features to further determine the prognostic indicators and is presented in Figure 2. In addition to some clinical parameters that are commonly thought to be closely associated with the prognosis of IgAN (proteinuria and eGFR), some newly discovered risk factors (hematuria, age and sex) whose prognostic efficacy is currently not widely accepted were also added. Notably, no significant difference was observed between patients in proteinuria < 1 g/d and proteinuria ≥ 1 g/d group with respect to kidney endpoint probability (*p* = 0.452), but patients with hematuria seemed to be more likely to progress to ESKD (*p* = 0.05) than patients with the absence of hematuria, although it was only marginally significant. The kidney survival rate was remarkably higher in patients with eGFR > 60 mL/min/1.73m2 than those in patients with eGFR ≤ 60 mL/min/1.73m2 (*p* = 0.005), suggesting that decreased kidney function at diagnosis has a negative impact on the outcome of IgAN. Similarly, patients aged over 60 years had a significantly higher kidney endpoint probability than quinquagenarian patients aged 50 to 60 years (*p* = 0.039), suggesting that older age might still serve as a predictor for adverse outcomes in elderly patients. Kaplan-Meier analysis also indicated better kidney survival in female patients than in male patients (*p* = 0.005).

### Differences in clinicopathological manifestations grouped by age and sex

We further explored clinicopathological differences according to sex and age in search of the possible reasons why age and sex were closely linked to kidney survival. The detailed features are shown in Table 3. Among these subjects, 87 patients aged 50 to 60 years were assigned to the quinquagenarian group, while 39 patients aged over 60 years were assigned to the geriatric group. At baseline, patients in the geriatric group presented with significantly lower albumin (34.12 ± 6.82 vs. 38.03 ± 6.22, *p* = 0.002), eGFR (57.99 ± 25.21 vs. 70.39 ± 26.32, *p* = 0.015), hemoglobin (122 [112–139] vs. 131 [120–145], *p* = 0.042), and aspartate aminotransferase (9.41 ± 1.51 vs. 8.80 ± 0.94, *p* = 0.026) than those in the quinquagenarian group. However, there were no significant differences between the two groups with respect to kidney pathologic lesions.

**Table 3.** Clinicopathological manifestations of the older IgAN patients at baseline, grouped by age and gender.

Variables	Age			Gender		
	Quinquagenarian patients 50–60 years old	Geriatric patient*s* ≥60 years old	*P*	Male	Female	*P*
Numbers (%)	87	39		65	61	
Age at diagnosis (years)	53.54 ± 2.99	65.10 ± 3.63	<0.001***	57.09 ± 6.69	57.15 ± 5.79	0.961
SBP (mmHg)	132.74 ± 16.89	135.97 ± 18.99	0.340	133.82 ± 18.38	133.66 ± 16.78	0.96
DBP (mmHg)	83.76 ± 11.56	85.87 ± 14.20	0.379	83.66 ± 14.03	85.21 ± 10.49	0.486
Hypertension (%)	29 (33.3)	6 (15.4)	0.052	50 (76.9)	41 (67.2)	0.239
Proteinuria (g/24h)	1.87 (1.00–3.00)	2.39 (1.00–4.08)	0.079	2.60 ± 2.32	2.82 ± 3.13	0.648
U-RBC (/HP)	20 (4–69)	32 (9–183)	0.096	107.25 ± 225.97	115.69 ± 216.79	0.831
Albumin (g/L)	38.03 ± 6.22	34.12 ± 6.82	0.002**	36.61 ± 6.82	37.05 ± 6.50	0.711
SCr (umol/L)	108.97 ± 62.56	120.48 ± 58.31	0.332	128.05 ± 74.12	96.00 ± 37.73	0.003**
e-GFR (ml/min/1.73m^2^)	70.39 ± 26.32	57.99 ± 25.21	0.015*	67.44 ± 27.48	65.61 ± 25.64	0.701
UA (umol/L)	402.30 ± 97.85	374.49 ± 97.77	0.143	419.51 ± 93.06	366.18 ± 96.94	0.002**
Hemoglobin (g/L)	131 (120–145)	122 (112–139)	0.042*	138 (120–151)	123 (114–135)	<0.001***
Anemia (%)	9 (10.3)	6 (15.4)	0.552	4 (6.2)	11 (18.0)	0.054
Triglyceride (mmol/L)	2.27 ± 1.52	1.78 ± 0.84	0.062	1.67 (1.12–2.19)	1.99 (1.40–2.86)	0.019*
Cholesterol (mmol/L)	5.23 ± 1.30	5.65 ± 1.26	0.089	5.06 ± 1.19	5.68 ± 1.34	0.007**
ALT (IU/L)	20 (14–27)	20 (16–35)	0.371	26.37 ± 13.08	20.49 ± 12.79	0.012*
AST (IU/L)	8.80 ± 0.94	9.41 ± 1.51	0.026*	25.31 ± 8.69	24.05 ± 9.61	0.442
M0/M1 (%)	21/66 (24.1/75.9)	6/33 (15.4/84.6)	0.350	9/56 (13.8/86.2)	18/43 (29.5/70.5)	0.049*
E0/E1 (%)	81/6 (93.1/6.9)	35/4 (89.7/10.3)	0.498	61/4 (93.8/6.2)	55/6 (90.2/9.8)	0.521
S0/S1 (%)	37/50 (42.5/57.5)	17/22 (43.6/56.4)	1.000	26/39 (42.9/57.1)	28/33 (45.9/54.1)	0.590
T0/1/2 (%)	73/14/0 (83.9/16.1/0)	32/6/1 (82.0/15.4/2.6)	0.439	51/13/1 (83.3/15.9/0.8)	54/7/0 (88.5/11.5/0)	0.185
C0/1/2 (%)	66/18/3 (75.9/20.7/3.4)	31/7/1 (79.5/17.9/2.6)	0.992	48/14/3 (73.9/21.5/4.6)	49/11/1 (80.4/18.0/1.6)	0.586

*SBP:* systolic blood pressure; *DBP:* diastolic blood pressure; *U-RBC:* the count of uric red blood cell; *SCr:* serum creatinine; *e-GFR:* estimated glomerular filtration rate; *UA:* uric acid; *ALT:* alanine aminotransferase*;* *AST:* aspartate aminotransferase*;* *M:* mesangial proliferation; *E:* endocapillary proliferation; *S:* segmental glomerulosclerosis; *C:* crescents; *T:* tubular atrophy/interstitial fibrosis.

*Stands for *p* < 0.05; **stands for *p* ≤ 0.01; ***stands for *p* ≤ 0.001.

Comparison between males (*n* = 65) and females (*n* = 61) indicated that male patients tended to have a higher level of hemoglobin (123 [114–135] vs. 138 [120–151], *p* < 0.001) and ALT (20.49 ± 12.79 vs. 26.37 ± 13.08, *p* = 0.012), as well as a lower level of TG (1.99 [1.40–2.86] vs. 1.99 [1.40–2.86], *p* = 0.019) and cholesterol (5.68 ± 1.34 vs. 5.68 ± 1.34, *p* = 0.007). Notably, although male patients had a higher concentration of SCr (96.00 ± 37.73 vs. 128.05 ± 74.12, *p* = 0.003), the difference in eGFR level between them was not distinctly significant. In addition, there were no significant intergroup differences in kidney pathologic lesions except that the male patients had a higher proportion of mesangial proliferation (29.5%/70.5% vs. 13.8%/86.2%, *p* = 0.049).

### The efficacy of immunosuppressive treatment in elderly patients with IgAN

**Figure 3.**
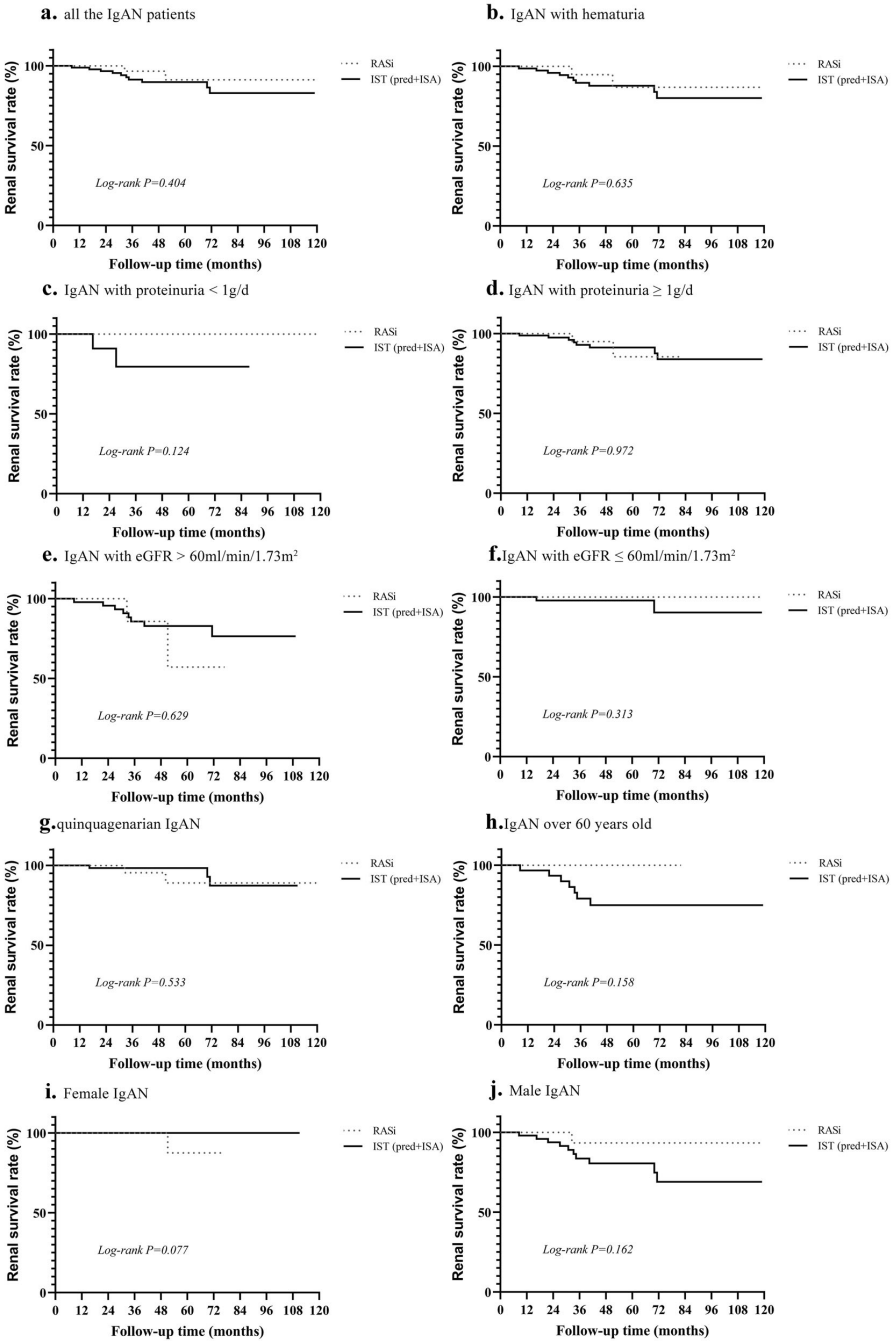
Kaplan-Meier curves of renal survival according to immunosuppressive treatment categories. (a) All the IgAN patients; (b) IgAN with hematuria; (c) IgAN with proteinuria < 1g/d; (d) IgAN with proteinuria ≥ 1g/d; (e) IgAN with eGFR > 60ml/min/1.73m^2^; (f) IgAN with eGFR ≤ 60ml/min/1.73m^2^; (g) quinquagenarian IgAN; (h) IgAN over 60 years old; (i) Female IgAN; (j) Male IgAN. IgAN, immunoglobulin A nephropathy; RASi, renin-angiotensin system inhibitors; IST, immunosuppressive therapy; Pred, prednisone; ISA, immunosuppressant.

To compare the therapeutic efficacy of RASi and IST in older IgAN patients, two groups were divided according to their treatment options, and a Kaplan-Meier survival curve analysis was performed (Figure 3). However, there was no significant difference in kidney outcomes between the RASi and IST groups, implying that the therapeutic efficacy of these two treatments did not differ in older IgAN patients (*p* = 0.404, Figure 3a). Moreover, subgroup analysis stratifying by levels of hematuria, proteinuria, eGFR, age, and sex was conducted, given that the efficacy of the therapies might be influenced by the differences in baseline characteristics (Figure 3). The results revealed that kidney endpoint rates between the two groups did not differ significantly regardless of subgrouping by the above parameters (*p* > 0.05), illustrating that immunosuppressive therapy might not be suitable for elderly IgAN patients.

## Discussion

IgAN occurs more frequently in young people aged 20–50 and is considered less common in elderly individuals. As the global population aged, IgAN progressively increased among the elderly during the last 25 years [22]. Literature regarding characteristics, treatment and outcomes in this age group did not correspond with this dramatic change. We then hereby reported relevant data in the elderly patients (age ≥ 50) with primary IgAN. A total of 126 patients diagnosed were finally recruited, with females accounting for 61%.

Age turned out to be an independent risk factor for poor kidney outcomes. Patients are grouped based on age to determine a possible explanation. Albumin and eGFR at baseline differed significantly between the quinquagenarian (50–60 years old) and geriatric groups (≥60 years old). Interestingly, pathological lesions based on Oxford Calcification between these two groups did not have significant differences. It is plausible to think that older patients had more severe kidney lesions at onset, which led to age-related poor prognosis. Similar tendencies were also observed in other studies. A retrospective single-center study conducted in the USA demonstrated that elderly patients with IgAN showed a higher risk of progression to ESKD than their younger counterparts [23]. Research based on 151 patients with IgAN (≥ 65 years old) from twenty-three centers also confirmed our result [22]. In contrast, in a study conducted by Wen et al., no significant difference in cumulative survival time was observed in IgAN according to age [8]. It was also reported that although elderly patients tended to have more severe histological lesions, aging was not an independent risk factor [24]. Of note, all patients in our study were over 50 years old, while the subjects of most research were across all age groups, which might explain the discrepancies among these studies. Furthermore, in addition to the proportions of elderly patients and the definition of old age, study design, lengths of follow up and outcome definition might also lead to conflicting results among these studies.

Male gender was another independent risk factor identified by our study. High serum creatinine, alanine aminotransferase and uric acid levels were found to be more common among male patients than among their counterparts. These findings were consistent with previous research [25,26]. A study by Deng et al. reported higher serum creatinine levels in males than in females with IgAN [25]. In another study, Wen et al. showed that male was positively associated with higher levels of serum creatinine and uric acid [26]. These findings indicated that male patients may have a higher risk of progression to unfavorable kidney outcomes, which may be mediated by worse clinical characteristics. Other studies held the same view that the prognosis of IgAN was totally different between males and females. Previously, several studies have demonstrated that the male gender was an independent risk factor for poor kidney prognosis according to Cox multivariable analysis [26]. We also found consistent results for IgAN in the male gender, conferring no long-term benefit over kidney prognosis. Related evidence suggests that estrogen might be partially responsible for better kidney outcomes in females [27]. The possible mechanism might be related to the expression of estrogen receptors in kidney tissue, which play a role in kidney damage and prognosis [28]. The sex difference in prognosis is also likely due to an unhealthy lifestyle in men [27]. However, the impact of sex on IgAN progression to ESKD remains controversial. Donadio et al. reported that the female gender, rather than the male gender, was associated with the progression of ESKD [29]. In a study performed by Deng et al., the male gender lost its prognostic effect after adjusting for baseline eGFR and serum uric acid levels in multivariable analysis [25]. In addition, Cattran et al. found no gender-related difference in long-term kidney outcomes [30]. The lack of agreement among these studies can be attributed to several factors, including the patient population, the mixed nature of the studies included, the length of follow-up, biopsy practice and different outcome definitions. In addition, O'Shaughnessy et al. proposed that differences in genetic and regional backgrounds might in part cause conflicting results [31]. Collectively speaking, we supported that male elderly patient tended to have worse kidney outcomes, and future multicenter studies with large sample sizes are needed to confirm this.

In our results, we also demonstrated that decreased kidney function and hypertension were associated with poor kidney outcomes among elderly patients with IgAN. In previous studies, the kidney outcomes of IgAN have conclusively been shown to be impacted by proteinuria, kidney impairment, anemia, and hypertension [6,32,33]. However, as for proteinuria and anemia, no significant association was observed. A study by Sevillano et al. reported that proteinuria was not significantly associated with poor outcomes, which was consistent with our findings [22]. It is suggested that clinicians should pay less attention to proteinuria in elderly patients, but prospective trials are still needed to determine this. In addition, elderly patients with hematuria had a higher risk of progression to adverse kidney outcomes, which was also inconsistent with most of the previous findings [11,32,34]. Although hematuria is a typical presentation of IgAN, its long-term prognostic value remains under debate. A recently published meta-analysis of 7 studies investigated the association between hematuria (binary variable) and ESKD [35]. They reported that initial hematuria was not associated with a significant increase in the risk of ESKD [35]. Of note, the average age of patients ranged from 30 to 45 in the meta-analysis, which may explain the discrepancy [11]. Additionally, hematuria analysis can also confuse results among studies since only one urine sediment measurement was used during analysis [10]. In addition, the number of erythrocytes in urine was variable in the short term [10]. Given the above evidence, time-averaged hematuria during follow-up may be worth attention and more data are needed to identify the prognostic value of hematuria among elderly patients with IgAN.

Another key finding in our study is that additional immunosuppressive therapy (Pred and ISA) was not superior to supportive treatment alone (RASi) in terms of therapeutic efficacy among IgAN patients at an advanced age. Immunosuppressants are an alternative choice for IgAN recommended by the KDIGO guidelines, but definitive conclusions regarding treatment efficacy have not been drawn [12]. The STOP-IgAN trial found that the additional immunosuppressive therapy did not confer renal benefits, which is consistent with our results [15]. Moreover, the trial also noted that adverse effects due to general immunosuppression could not be neglected. However, in the TESTING trial, the renal benefit of corticosteroids was observed, but due to early termination, the researchers failed to provide conclusive evidence [17]. Regarding side effects, they also highlighted safety concerns about steroids, consistent with STOP-IgAN [17]. Whether IgAN patients have an overall favorable response to immunosuppressants remains controversial, and most studies only included patients ranging from 20 to 40, without focusing on the elderly population [13,15–17]. Therefore, evidence regarding the treatment response to immunosuppressants in elderly IgAN patients is remarkably scarce, and only a few retrospective studies have been reported for elderly patients [22,23]. In a study conducted by Cheung et al., no significant difference was observed between elderly patients with and without steroid treatment in terms of kidney survival [23]. Another study by Sevillano et al. demonstrated that immunosuppressants were not associated with better kidney outcomes in elderly patients with IgAN [22]. Due to decreased immune reactivity, elderly patients are more susceptible to drug-related adverse effects, particularly cardiovascular diseases and infection [36], which have been shown to negatively influence kidney transplant patient survival [37]. Taken together, these results indicated that treatment with immunosuppressants might confer no overall benefit for elderly IgAN patients, who are more susceptible to adverse effects. Nevertheless, few data regarding elderly IgAN are available, so prospective controlled trials are needed to determine the efficacy of immunosuppressants in older patients with IgAN.

In Japan, tonsillectomy combined with corticosteroid pulse therapy is recommended for younger IgAN patients [38]. However, to the best of our knowledge, no clinical study has investigated the safety and effect of tonsillectomy for elderly IgAN patients [24]. In our department, tonsillectomy was not performed in elderly patients, because tonsillectomy might cause serious complications, such as infection due to immunosuppression, and the effect on elderly patients remains controversial. Recently, a letter by Takashima et al. reported that tonsillectomy is relatively safe and may improve kidney outcomes among elderly IgAN patients [39]. However, this conclusion was not convincing due to the short observation period and the small sample (14). Large prospective studies should be performed in the future.

There are several limitations to our study. First, the nature of the study was observational, which induced unavoidable selection bias. Second, the follow-up period was relatively short, whereas IgAN is an indolent disease. Third, our findings may not be applicable to IgAN patients from other regions, since the patients in our research were almost Chinese. Fourth, the sample size was relatively small, which might have not truly reflected the target population. In addition, some unmeasured confounding factors that could have an impact on kidney outcomes cannot be entirely considered.

In conclusion, male patients with advanced age and hematuria have a higher risk of progression to unfavorable outcomes. More aggressive diagnostic approaches should be considered for patients with these characteristics to capture the disease at an earlier stage. In addition, the therapeutic efficacy of immunosuppressive therapy and RAAS blockade did not differ significantly in elderly patients. Investigation of new therapeutic alternatives for elderly patients is urgently needed.

## Ethics Statement

All the authors have read and approved the manuscript and the requirements for authorship. Each author believes that the manuscript represents honest work.

### Trial registration

TCTR20180313003, Thai Clinical Trials Registry (No. TCTR20180313003, https://www.thaiclinicaltrials.org/show/TCTR20180313003).
